# White Matter Correlates of Musical Anhedonia: Implications for Evolution of Music

**DOI:** 10.3389/fpsyg.2017.01664

**Published:** 2017-09-25

**Authors:** Psyche Loui, Sean Patterson, Matthew E. Sachs, Yvonne Leung, Tima Zeng, Emily Przysinda

**Affiliations:** ^1^Music, Imaging and Neural Dynamics Lab, Department of Psychology, Program in Neuroscience and Behavior, Wesleyan University, Middletown CT, United States; ^2^Department of Psychology, Brain and Creativity Institute, University of Southern California, Los Angeles CA, United States; ^3^The MARCS Institute for Brain, Behaviour and Development, Western Sydney University, Penrith NSW, Australia

**Keywords:** music, evolution, auditory, affective, communication, diffusion tensor imaging

## Abstract

Recent theoretical advances in the evolution of music posit that affective communication is an evolutionary function of music through which the mind and brain are transformed. A rigorous test of this view should entail examining the neuroanatomical mechanisms for affective communication of music, specifically by comparing individual differences in the general population with a special population who lacks specific affective responses to music. Here we compare white matter connectivity in BW, a case with severe musical anhedonia, with a large sample of control subjects who exhibit normal variability in reward sensitivity to music. We show for the first time that structural connectivity within the reward system can predict individual differences in musical reward in a large population, but specific patterns in connectivity between auditory and reward systems are special in an extreme case of specific musical anhedonia. Results support and extend the Mixed Origins of Music theory by identifying multiple neural pathways through which music might operate as an affective signaling system.

## Introduction

Music is celebrated and valued in every human culture, yet we know relatively little about why music exists, or what functions music might serve for humankind. The evolutionary function of music has been a subject of debate since Darwinian times (Darwin, 1871). On the one hand, some scholars espouse views that music is an evolutionary byproduct that confers no cognitive advantage, i.e., that music is “auditory cheesecake” (Pinker, 1997). On the other hand, most researchers in the field of music perception and cognition posit that music serves many adaptive functions (Huron, 2001; Honing et al., 2015). For each of these functions, musical sounds function as an auditory channel for interpersonal communication, possibly preceding speech and language (Mithen, 2007). Thus, the need for interpersonal communication through an auditory channel is at the core of evolutionary pressures that are thought to shape music.

This need for interpersonal communication likely changes the cognitive system by virtue of lasting effects that music exerts upon our species. The notion that music is a human invention but transforms our experience, i.e., that music is a transformative technology of the mind (TTM) (Patel, 2008, 2010), is attractive for two reasons. Firstly, the TTM view reconciles the debate between more traditional adaptationist and exaptationist views (cf. Justus and Hutsler, 2005; Trainor, 2006) by pointing out the false dichotomy between these two views. Secondly, TTM brings to the forefront the idea that brains can change as a result of musical experience. Thus, studies that relate musical experience to inter-individual variability within our species may be informative of how music came to be valued in our species. Another important evolutionary role of music is in its value as an emotional signal: music has power to communicate and evoke strong emotions through an auditory channel (Snowdon et al., 2015). Building on to Patel’s TTM theory, the Mixed Origins of Music (MOM) theory posits that music transforms the brain through an affective signaling system that is common to many socially living animals (Altenmüller et al., 2013b; Snowdon et al., 2015). Specifically, the neural mechanisms through which chills occur in response to music may be informative of the evolution of music as an affective communication tool (Altenmüller et al., 2013b). Music elicits a variety of emotions: from abstract, aesthetic experiences to strong, more physiologically measurable emotional responses (Scherer, 2004). Several underlying mechanisms have been proposed through which emotional responses to music might be elicited. For instance, the BRECVEMA model provides a comprehensive account of emotional mechanisms for music (Juslin and Västfjäll, 2008; Juslin, 2013); they include (among others) brainstem responses and evaluative conditioning mechanisms, which involve brain areas within the dopaminergic reward system.

Music that is rewarding is processed by functional connectivity between auditory areas [superior temporal gyrus (STG)] and reward system areas such as the nucleus accumbens (NAcc) (ventral striatum), caudate (dorsal striatum), and areas in the classic limbic system including the amygdala and anterior insula (AIns) (Salimpoor et al., 2011, 2013). Individual differences in the tendency to derive chills, i.e., measurable psychophysiological responses, from music are associated with structural connections from auditory regions (STG) to the AIns, which is consistently activated during the experience of strong emotions, and the medial prefrontal cortex (mPFC), which is important for computing social value; furthermore, this association is modulated by connectivity through the NAcc, a hub in the dopaminergic reward system (Sachs et al., 2016). On the extreme end of the spectrum of individual differences in musical reward, recent work has found evidence for specific musical anhedonia, a rare but intriguing condition where individuals derive no reward responses from their musical experience (Mas-Herrero et al., 2013, 2014). The underlying brain mechanisms are similar to those reviewed above, in that they involve functional connectivity between auditory regions and reward regions, notably the dopaminergic pathway centering around the NAcc (Martínez-Molina et al., 2016).

Although differences in functional connectivity and general brain structure have both been observed in subjects with musical anhedonia (Martínez-Molina et al., 2016; Belfi et al., 2017), it is not yet known whether, and to what extent, these functional connectivity differences identified in musical anhedonics might also be structurally detectable. Furthermore, although structural brain differences in white matter connectivity between auditory and emotion and reward areas have been related to individual differences in reward responses to music (Sachs et al., 2016), it is unknown whether specific musical anhedonia simply reflects the low end of a continuum of normal individual differences in brain connectivity and reward responses to music, or whether musical anhedonia is a categorically distinct disorder that reflects anatomically dissociated neural substrates from normal variations in reward sensitivity. If the former is the case (i.e., musical anhedonia represents the low end of a single continuum), then one would expect that differences in auditory-to-reward connectivity between musical anhedonics also extend to the rest of the population. Conversely, if the latter is the case (i.e., musical anhedonia is different from normal variability in musical reward), one would expect that musical anhedonics have different patterns of reward and auditory-to-reward connectivity from the variations that are generally observed within the population that reflect reported differences in the experience of reward from music.

Here we test the primary hypothesis that musical anhedonia reflects specific differences in white matter connectivity within the reward system, and between the auditory and reward systems. Secondly, we test the hypothesis that the same patterns of white matter connectivity reflect individual differences in the normal variations of reward experiences in music. Using combined behavioral and diffusion tensor imaging (DTI) methods, we compare the white matter connectivity of a musically anhedonic subject, BW, to a group of normal controls (*n* = 46) who report a range of reward from music. Results will identify the neuroanatomical networks that predispose the human brain toward successful affective communication through music.

## Materials and Methods

### Subjects

Subject BW (male, age 53 years, right-handed) presented with a self-reported, socially debilitating lack of reward experience from music despite intact reward responses to visual art. **Table 1** shows demographic information and information about musical training. Screening measures including Montreal Battery for Evaluation of Amusia (Peretz et al., 2003) and the nonverbal measure of the Shipley Institute of Living Scale (Shipley, 1940) were used to rule out any differences due to amusia or general intellectual impairment, respectively.

**Table 1:** Demographic information, baseline tests, and scores on Barcelona Music Reward Questionnaire and Physical Anhedonia Scale for BW and control subjects.

	Control group; mean (SD)	BW
*N*	46	1
Number of females	17	N/A
Age	20.5 (4.66)	53
Years of musical training	7.30 (4.44)	4
Age of onset of musical training	8.35 (2.98)	13
Shipley score	17.2 (1.91)	15
MBEA (% correct)	81.4% (6.83%)	80.6%
**BMRQ scores**		
Musical reward	49.0 (11.3)	-9
Music seeking	53.4 (11.4)	13
Emotional evocation	46.2 (13.3)	2
Mood regulation	49.0 (12.2)	-1
Sensorimotor	44.3 (10.6)	12
Social reward	55.4 (11.9)	24
**PAS (% anhedonic score)**		
Sound items	19.6% (13.7%)	90.9%
Non-sound items	16.7% (11.2%)	21.7%

Control subjects (*n* = 46, 17 females, all right-handed) consisted of Wesleyan students and community members. Subjects reported a variety of musical training, and tested within normal ranges for MBEA and Shipley (**Table 1**). Among the control subjects, 85% (39 subjects) completed the BMRQ. All subjects gave written informed consent as approved by the Institutional Review Boards of Wesleyan University and Hartford Hospital.

### Stimuli

In addition to screening tools reported above, 39 of the 46 subjects completed the Revised Physical Anhedonia Scale (PAS) (Chapman et al., 1976) and the Barcelona Music Reward Questionnaire (BMRQ) (Mas-Herrero et al., 2013). The PAS is a self-report scale used to measure anhedonia, the lowered ability to experience pleasure (Chapman et al., 1976). It consists of 61 statements that describe pleasurable experiences (e.g., “I have usually found lovemaking to be intensely pleasurable.”). Subjects are asked to indicate whether each statement is true or false as it applies to them. Among the 61 statements, 10 items pertain to sounds (e.g., “The sounds of a parade have never excited me.”) whereas the others are non-sound items that include other sensory and social pleasures (e.g., “I have often found walks to be relaxing and enjoyable.” “I have often enjoyed receiving a strong, warm handshake.”).

The BMRQ (Mas-Herrero et al., 2013) was used to assess how BW experienced reward associated with music, in comparison with the control group. The BMRQ is a 20-item questionnaire designed to measure musical reward experiences as a combination of five factors: musical seeking, emotion evocation, mood regulation, sensory-motor, and social reward.

### Procedures

After informed consent procedures, subjects completed surveys to report their demographic and musical training data. They also completed the MBEA and Shipley tests as screening measures for amusia and intellectual impairment. They then completed the PAS and BMRQ to assess possible general and musical anhedonia.

In addition to behavioral data, high-resolution T1 and DTI images were acquired in a 3T Siemens Skyra MRI scanner at the Olin Neuropsychiatry Research Center at the Institute of Living. Anatomical images were acquired using a T1-weighted, 3D, magnetization-prepared, rapid-acquisition, gradient echo (MPRAGE) volume acquisition with a voxel resolution of 0.8 mm × 0.8 mm × 0.8 mm. Diffusion images were acquired using a diffusion-weighted, spin-echo, echo-planar imaging sequence (TR = 4.77 s, voxel size = 2.0 mm × 2.0 mm × 2.0 mm, axial acquisition, 64 noncollinear directions with *b*-value of 1000 s/mm^2^, 64 noncollinear directions with *b*-value of 2000 s/mm^2^, 1 image with *b*-value of 0 s/mm^2^).

### Data Analysis

All MR images were processed using FMRIB’s Software Library (FSL) (Jenkinson et al., 2012). The images were then corrected for eddy current distortions using the eddy correct function. Non-brain structures were removed from each participant’s images by the brain extraction tool. A diffusion tensor model was fit at each voxel in the extracted brain using the dtifit function to get a fractional anisotropy (FA) image for each participant. Probabilistic tractography was conducted using a Bayesian Estimation of Diffusion Parameters Obtained using Sampling Techniques (bedpostX) to determine the probable directions of each fiber for each brain voxel (Behrens et al., 2007).

Probabilistic tractography was conducted to determine structural connectivity in each hemisphere between each pair of the following regions of interest: STG, AIns, mPFC, and NAcc. The same regions were used as in our previous study (Sachs et al., 2016), as they were specifically identified to include white matter regions within the reward system (mPFC, NAcc, and aIns) and the auditory system (STG). The STG and NAcc were extracted from the Harvard-Oxford Cortical atlas (Desikan et al., 2006), and masked with a standardized FA image. The AIns was extracted from the LONI atlas (Shattuck et al., 2008). Then, using previous literature as a reference (Uddin and Menon, 2009), the anterior portion was defined anatomically within the lateral sulcus. As atlases varied in their delineation of the prefrontal cortex, the mPFC was hand drawn on coronal slices in the anterior portion of the corona radiata (Marchina et al., 2011). Each ROI was extracted or hand drawn on the standardized FA template by a first coder, and verified by a second coder. Each ROI was then warped to each individual subject’s FA image in native space and binarized.

Tractography was then initiated from each ROI as a seed toward each other ROI as a waypoint mask; and then tractography was initiated again using the original waypoint mask as the seed and the original seed as the waypoint mask; these two directions of probabilistic tractography were then averaged to yield a single tract between each pair of regions. Each resultant tract was averaged and then thresholded at 10% of its robust intensity level to minimize extraneous tracts. Tract volume and mean FA of the normalized tracts were exported for statistical comparisons. Additionally, to enable visualization all subjects’ tracts and FA images were aligned and normalized to the FSL 1 mm FA template using both linear registration (FLIRT) (Jenkinson and Smith, 2001) and nonlinear registration (FNIRT) tools, and canonical tract images were created by averaging each binarized tract across subjects in the control group, and thresholding voxels below the median.

One-sample *z*-tests were used to compare tract volume and normalized FA between BW and control group. Furthermore, to test for brain–behavior relationships within the control group, we ran two separate multiple regression models, both using Music Reward (overall score from the BMRQ) as the dependent variable. The first regression used FA values from each tract as predictor variables; the second regression used volumes from each tract as predictor variables. Collinearity for all variables in both regressions was minimal (Tolerance > 0.1, VIF < 8). For tracts that were significant predictors of the Music Reward score, we also conducted follow-up tests for correlations between tract FA and each of the five subscores from BMRQ, while applying the Bonferroni statistical correction for the five subscores.

## Results

### Behavioral Results

**FIGURE 1:**
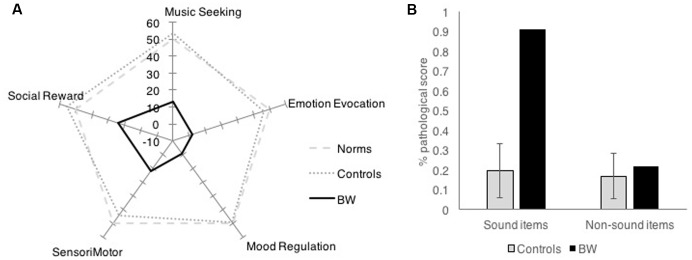
Survey results comparing BW against controls in **(A)** Barcelona Music Reward Questionnaire and **(B)** Physical Anhedonia Scale.

Barcelona Music Reward Questionnaire showed that BW had low reward response to music in all categories of musical reward. While controls had an average factor score of 50 (*SD* = 10) on the BMRQ (Music Reward overall score), BW had an overall factor score of -9, which was 5.89 standard deviations below controls. BW scored more than 2.5 standard deviations below controls on all subscales of the BMRQ (**Figure 1A**).

Physical Anhedonia Scale showed that BW was not generally anhedonic, except for items that pertain to sound. Control subjects generally scored an average of 17% of responses in the anhedonic (“pathological”) direction (*SD* = 9%). BW scored a total of 39% of responses in the anhedonic direction. Item analysis of the PAS was done by separately analyzing sound and non-sound categories. While the control subjects showed similar proportions of anhedonic scores for sound items and non-sound items (*M* = 19.6%, *SD* = 14% anhedonic responses for sound items; *M* = 16.8%, *SD* = 11% anhedonic responses for non-sound items); BW showed 21.7% anhedonic scores for non-sound items (within 1 SD of the mean) but 90.9% anhedonic responses for sound items (more than 5 SD above the mean). This striking dissociation (**Figure 1B**) suggests that BW does not have general anhedonia, but is specifically anhedonic toward sounds, especially to music.

### DTI Results

#### Musical Anhedonic vs. Controls

**FIGURE 2:**
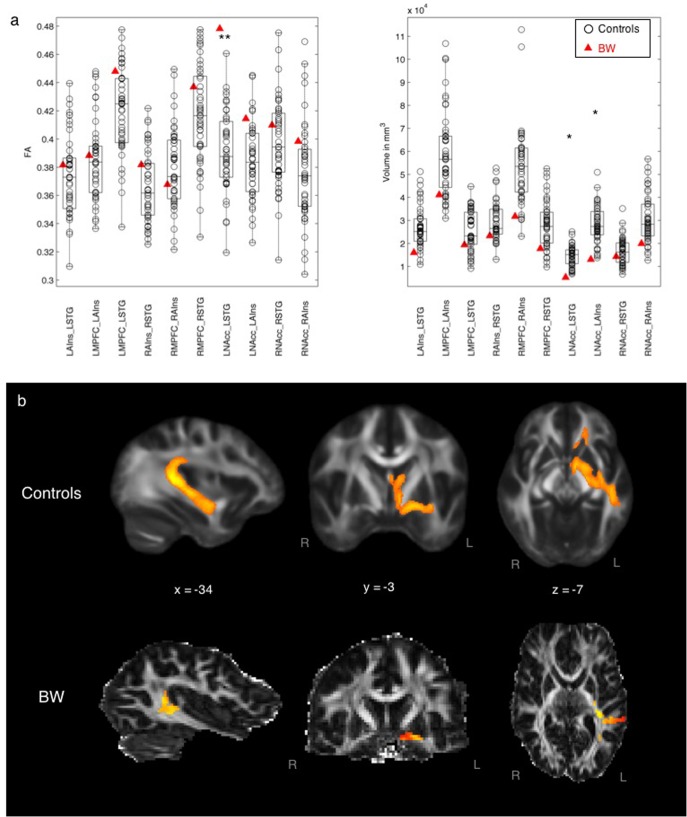
**(a)** Left: Mean FA of each tract comparing BW and controls, controlling for volume differences. ^∗∗^*p* < 0.05 Bonferroni-corrected (0.05/10 = 0.005). Right: Volume of each tract comparing BW and controls. ^∗^*p* < 0.05 uncorrected. **(b)** Averaged tract between STG and NAcc for controls (top row) and for BW (bottom row).

**Figure 2** compares tract FA and volume between BW and control subjects, showing some differences in auditory–reward connectivity in the subject with musical anhedonia. BW had significantly lower tract volume than controls in tracts between the left STG and left NAcc (*z* = -2.16, *p* = 0.03) and between the left AIns and left NAcc (*z* = -1.98, *p* = 0.04) at the uncorrected *p* < 0.05 level. No other tracts showed statistically significant differences between BW and controls according to *z*-tests. Mean FA (after normalizing for volume to enable a direct comparison of FA values) was greater for BW than controls between left STG and left AIns (*z* = 3.08, *p* = 0.002), the same tract in which he showed lower volume than controls, surviving Bonferroni correction at *p* < 0.05/10. No other tracts showed significant differences in FA according to the *z*-test.

#### Individual Differences within Control Group

**FIGURE 3:**
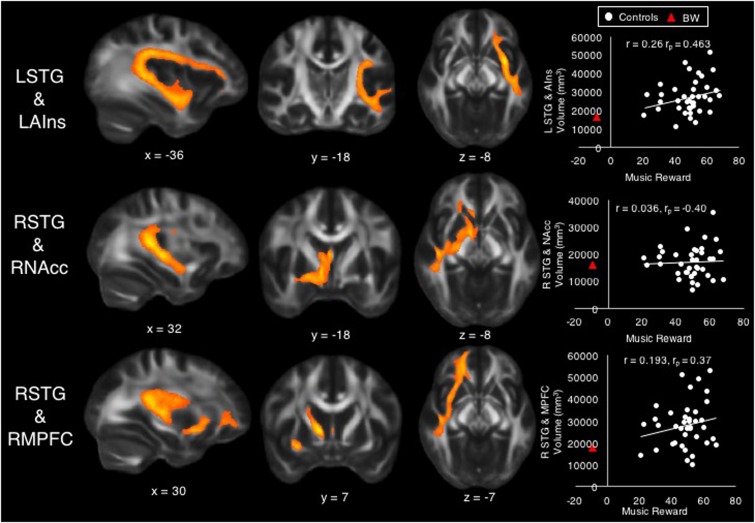
Volumes of tracts between left superior temporal gyrus (STG) and left anterior insula (top), between right STG and right nucleus accumbens (middle), and between right STG and right medial prefrontal cortex (bottom) were significant predictors of the Music Reward score among control subjects. Scatterplots show bivariate correlations (*r*) between Music Reward score and the volume of each tract, as well as partial correlation coefficients (*r*_p_) from the regression for purposes of comparison against bivariate correlations. BW’s data are also shown on scatterplots for purposes of comparison.

A multiple regression model with the dependent variable of Music Reward score, with tract volume (in mm^3^) of each tract as predictor variables, accounted for 38% of the variability (*R*^2^ = 0.38), but was not significant after accounting for the number of predictors (adjusted *R*^2^ = 0.15, *F* = 1.69, *p* = 0.13). Among the controls, the Music Reward score was significantly predicted by the volume of tracts between LSTG and LAIns (β = 1.11, *t* = 2.76, *p* = 0.01, bivariate correlation *r* = 0.26, partial correlation *r*_p_ = 0.463), between RSTG and RNAcc (β = -0.81, *t* = -2.33, *p* = 0.027, *r* = 0.036, *r*_p_ = -0.40), and between RSTG and RMPFC (β = 0.74, *t* = 2.10, *p* = 0.045, *r* = 0.193, *r*_p_ = 0.37). Although these tract volumes were significant predictors of Music Reward at the *p* < 0.05 level, they did not survive correction for multiple comparisons across the 10 tested tracts. **Figure 3** shows these tracts and scatterplots of their bivariate correlations with the Music Reward score.

A multiple regression model with the dependent variable of Music Reward score, with FA values of each tract as predictor variables, accounted for 26% of the variability (*R*^2^ = 0.26, adjusted *R*^2^ = -0.002, *F* = 0.99, *p* = 0.47). None of the tracts emerged as significant predictors (all *p* > 0.05).

#### Predicting Musical Anhedonic Brain and Behavior from Control Group Data

To assess whether BW falls along the same continuum of brain–behavior relationships as predicted by controls, we first used the regression model from all tract volume data to generate a prediction for BW’s Music Reward score. Given the multiple regression model obtained from tract volume data above (see section “Individual Differences Within Control Group”), BW’s tract volume data predicted his Music Reward score to be 0.29, which was much higher than his actual score (-9). However, pairwise correlations between behavior and tract volume (scatterplots in **Figure 3**) showed that BW is a predictable outlier from the control subjects’ data, with low volume in tracts between LSTG and LAIns and between RSTG and RMPFC, as predicted by his low Music Reward score and by control subjects’ data. To assess whether BW’s tract volumes belonged to the same continuum as controls, we used the slope and intercept of the trend line that best fit the bivariate relationship among control subjects to predict BW’s tract volumes using his Music Reward score (**Table 2**), thus extrapolating control subjects’ data to predict BW’s tract volumes. The prediction fits BW’s actual data with 7.6% error for LSTG_LAIns tract, with 9.4% error for the RSTG_RNAcc tract, and with 1.0% error for the RSTG_RMPFC tract, suggesting that for these three tracts, BW falls on the extreme end of the same continuum as the control subjects.

**Table 2:** Predicting musical anhedonic from control data.

Tract	Slope	Intercept	Prediction	Actual	Error^a^
Predicting BW’s music reward score from overall regression model			
All	5.23	47.36	43.58	-9	5.84
Predicting BW’s tract volume scores from bivariate correlation trend lines			
RSTG_RMPFC	178.49	19,204	17,597.59	17,776	0.010036566
LSTG_LAIns	206.02	16,768	14,913.82	16,040	0.070210723
RSTG_RNAcc	18.791	15,935	15,765.881	14,280	-0.1040533

*BW’s music reward score was not successfully predicted (103% error) from the overall regression model of DTI predictors. In contrast, BW’s tract volume of RSTG_RMPFC, LSTG_LAIns, and RSTG_RNAcc tracts was successfully predicted (10% error) from bivariate correlations between music reward and tract volume. ^a^[(Actual-Prediction)/Actual]*.

## Discussion

Individual differences in brain and behavior can be demonstrated by the normal variance within the general population, as well as extreme cases where substantial variations in brain and behavior give rise to striking deviations from the general population. To the evolution of music, the existence of musical anhedonia presents one such model of a striking dissociation, in which some individuals have a lack of reward responses specifically to sound. Here, we see that patterns of white matter connectivity in the auditory and reward systems reflect individual differences in the tendency to perceive reward from music. Auditory–reward connectivity differences are observed in our extreme case of musical anhedonia, and also reflect individual differences in music reward sensitivity within the control group.

BW, a subject with severe musical anhedonia, had decreased white matter volume but higher FA between auditory and reward areas, specifically between left STG and left NAcc. The left STG is a cortical hub of the auditory system: it includes auditory belt and parabelt areas which are important for analyzing temporal content of sounds, including speech-specific content (Overath et al., 2008, 2015). The NAcc is central to the mesolimbic pathway of the dopaminergic reward system, with its known role in reward and reinforcement (Wise, 2006), and is the crucial waystation of a reward network activated during the peak experience of music-related reward (Salimpoor et al., 2011; Zatorre and Salimpoor, 2013; Koelsch, 2014). Although the left NAcc showed higher volume of connectivity to the ipsilateral AIns as well as STG, the volume results were only significant at the uncorrected level; in contrast, the increased FA between left NAcc and STG was significant at the Bonferroni-corrected level in BW. FA, the main outcome variable in DTI, is an index of white matter integrity which includes myelination and coherence of axonal bundles. Probabilistic tractography requires FA values of each voxel to be above the white matter threshold, in order to derive tract volume (Behrens et al., 2007). Here, the pattern of simultaneously increased white matter integrity and decreased volume may suggest increased myelination and/or decreased crossing fibers in BW’s anatomical connections between LSTG and LNAcc, which could result in increased inhibition from LSTG to LNAcc. Functionally, the increased inhibition from LSTG could lead to a downregulation of the activity of LNAcc, resulting in deactivation of the NAcc as observed in recent functional MRI work in musical anhedonics (Martínez-Molina et al., 2016). Although these results are correlative rather than causal, the finding that BW had decreased volume but increased white matter integrity between these two regions adds to existing literature on the role of auditory–reward connectivity in affective responses for music (Salimpoor et al., 2013; Sachs et al., 2016); the implications of this data pattern for the evolution of music will be considered again later in this section “Discussion”.

The PAS showed that BW was anhedonic to all sound items, including non-music items (e.g., “the sounds of a parade”; “the cackling of fire in a fireplace”). Upon further interview, BW stated: “The crackle of a fireplace, the rustle of leaves, the swish of ocean waves – I just don’t appreciate them.” It remains to be seen whether musical anhedonics in other studies also report anhedonia toward sound items from the PAS, or whether BW is unique in his lack of appreciation of all auditory stimuli. If BW is different from other musical anhedonics in this regard, then one might expect that his auditory–reward disconnection is also more general than other cases of musical anhedonia.

Regarding the lack of appreciation for sounds, an interesting related question concerns whether BW could have misophonia, another auditory disorder where an individual reacts aversively to trigger sounds (Kumar et al., 2017). While more research is needed in the future to determine the extent of overlap or shared traits between misophonia and musical anhedonia, our study identifies BW as having musical anhedonia rather than misophonia, mainly because BW’s main complaint is that he feels no enjoyment from music, rather than being angered or anxious in response to trigger sounds as is common among misophonics (Edelstein et al., 2013). According to his self-report: “Music doesn’t particularly change my mood or give me an emotional response.” “Music never disgusts me. (The taste of cheese disgusts me. The smell of rotten eggs disgusts me. The sight of gore disgusts me.) Mostly I’d say that I’m neutral about music, because I just don’t care (and I don’t care that I don’t care!), and I mostly tune it out.” He also reports normal responses to speech and nonverbal vocal sounds. In contrast, misophonics most commonly report feeling disgusted, trapped, and/or anxious in response to trigger sounds, which are typically sounds produced by other people (Edelstein et al., 2013). From our findings, BW shows an abnormal pattern of connectivity from the NAcc; this was not observed in misophonics (Kumar et al., 2017). Thus, at present results suggest that musical anhedonia pertains more to a lack of reward, whereas misophonia pertains more to the experience of negative emotions such as anger and irritation in reaction to trigger sounds.

Within our control group, volume of some tracts between auditory and reward regions, specifically between LSTG and LAIns, between RSTG and RNAcc, and between RSTG and RMPFC, were predictive of musical reward at the 0.05 (uncorrected) level. Although these results do not survive correction for multiple comparisons, it is noteworthy that only tracts from left or right STG (the only auditory regions in our model) emerged as significant predictors, suggesting that individual differences in music reward do pertain to auditory-specific access to the reward system. It is also noteworthy that BW’s tract volume data can be predicted by extrapolating the trend line that best fits the bivariate relationship between music reward and volume of the significant predictor tracts. In contrast, the multiple regression model obtained from control subjects did not accurately predict BW’s music reward score. Thus, control subjects’ data can predict BW’s tract volumes but not his behavioral scores. This may be because BW’s music reward score, at 5.89 *SD* below controls, is much more of an outlier than his brain measures; thus, the brain predictors of behavior derived from control subjects do not apply to BW’s very unusual behavioral data, but BW’s tract volume data appear to lie at the low end of a normal distribution. The fact that BW is a very extreme outlier on the BMRQ also suggests that true musical anhedonia, at least as represented by the case of BW, is probably very rare. This is consistent with the observation that across patients of many types of brain damage, few report musical anhedonia (Belfi et al., 2017). Future studies might rely on more targeted strategies to identify more such cases of musical anhedonia.

The tract between LSTG_LAIns shows a continuum in volume that best reflects our range of behavioral data: its volume is reduced in the musical anhedonic as well as positively correlated with music reward. Connections between AIns and STG likely include the arcuate fasciculus, part of the auditory dorsal pathway that connects superior temporal and inferior frontal regions that is related to musical ability (Loui et al., 2009, 2011; Halwani et al., 2011; Loui, 2015). Furthermore, AIns is reduced in functional connectivity to auditory cortex in singers (Kleber et al., 2013), and functional connectivity between LSTG and LAIns is correlated with lexical retrieval in spontaneous speech (Chai et al., 2016). In addition to its role in vocal–motor integration and speech, the AIns is part of the classic limbic system and is implicated in the quartet theory of emotions due to its importance in interoception and emotional regulation (Koelsch et al., 2015). Given these diverse roles of AIns in the auditory–motor system, the present finding of increased tract volume between left AIns and LSTG in controls who experience high musical reward may relate to auditory–motor behavior especially as it applies to vocal–motor behavior. This auditory–insula connectivity may be related to the differentiation of vocalization repertoire as posited in the MOM theory (Altenmüller et al., 2013a). The MOM theory states that differentiation of vocalization repertoire, as driven by chill experiences, led to the capacity for fine-grained rhythmic–melodic discrimination. In our evolutionary history, it is possible that individuals with high LSTG_LAIns connectivity, who were highly reward-sensitive to music (e.g., frequently experiencing chills in response to music), then went on to acquire fine-grained auditory discrimination skills, which then gave rise to language and music. Since the AIns is an evolutionarily older part of the brain than its neighbor the inferior frontal gyrus (which is a classic endpoint of the arcuate fasciculus) (Galaburda and Pandya, 1982; Semendeferi and Damasio, 2000), the LSTG_LAIns connection could have predated the arcuate fasciculus, thus serving as a pathway for the differentiation of vocalization response that preceded the hypothesized bifurcation of auditory information into music and language (Mithen, 2007).

Superior temporal gyrus connections to NAcc and mPFC may include the arcuate as well as the uncinate fasciculus, the latter being part of the auditory ventral pathway that connects the temporal and frontal lobes (Wakana et al., 2004) and is involved in processing local syntactic structures (Friederici, 2009). mPFC is also part of the default mode network and is involved in social, self-referential, and emotional processing (Fox et al., 2005; Mason et al., 2007; Jenkins and Mitchell, 2010; Kim and Johnson, 2015). As the mPFC is a waystation of the dopaminergic system that probably emerged later in evolution (Galaburda and Pandya, 1982; Semendeferi and Damasio, 2000), the finding that connections to it correlate with musical reward suggests a further involvement of an evolutionarily younger part of the dopaminergic system in music processing beyond the NAcc. Interestingly, while the LSTG_LAIns and LSTG_LMPFC tracts show positive bivariate as well as significantly positive partial correlations to music reward, the RSTG_RNAcc tracts show no significant bivariate correlation with music reward, but a significant negative partial correlation after partialling out the effects of the other predictors. This is especially intriguing when considered alongside data from the musical anhedonic subject: BW had a lower volume but higher FA in LSTG_LNAcc; highly hedonic controls had lower volume in RSTG_RNAcc. Together these results suggest that auditory access to the mesolimbic pathway is hemispherically asymmetric, with normal variations in reward sensitivity occurring on the right but abnormal lack of reward on the left. This is consistent with hemispheric asymmetry to attractive vs. aversive stimuli in animals, but only in learned responses (Besson and Louilot, 1995; Molochnikov and Cohen, 2014). In light of the MOM theory, which posits that chill responses were initially a reward to novel auditory patterns prior to its driving of differentiated vocalization repertoire as discussed above, the present findings link the STG_NAcc pathway to this very early step in the evolution of music.

While this study cannot tease apart when or how these individual differences emerged, the pattern of results can be considered in the context of known steps in brain evolution as well as development, which together provide support for the MOM theory. Our rare case of musical anhedonia possesses a different pattern of white matter pathways between auditory regions and reward-sensitive regions, possibly due to abnormal neuronal migration *in utero* or early in development. In the multiple regression analysis to predict musical reward scores from diffusion measures, since we tested pairwise connections between regions in the auditory and reward networks, this necessarily resulted in an elevated number of statistical comparisons. The brain–behavior relationships within the control group are only significant at the uncorrected level. Thus, although the current results are interesting they should be interpreted cautiously until further verification. Nevertheless, the FA difference between BW and the control in the LSTG–NAcc tract survives correction for comparisons across the 10 tested tracts; this gives us higher confidence in a structural difference between auditory and reward areas that is linked to musical anhedonia.

A remaining question concerns whether musical anhedonia is likely to be a spectrum disorder. The answer to this question depends on how we define musical anhedonia. Considering that the BMRQ is for now the only diagnostic tool explicitly in use to identify musical anhedonia, and it yields a continuum of scores when administered to a large population (Mas-Herrero et al., 2013), the lack of musical reward appears to be continuously distributed. On the other hand, if we define musical anhedonia by self-identification of a socially debilitating lack of reward experiences specific to music, then it might not be a spectrum. However, defining musical anhedonia by self-identification would mean that identification depends upon the subject’s awareness of their own condition, which would in turn depend on their social environment. For instance, if BW had not heard about musical anhedonia, or if he lived in an environment where music was less celebrated, then he might not have become aware of his condition. Thus, large-scale testing of musical reward sensitivity across different cultures may be helpful for future definitions of cultural norms against which we define musical anhedonia.

Results show that musical anhedonia is related to different patterns of connectivity from auditory to emotion and reward centers of the brain. This auditory access to the reward system informs the evolutionary basis of music: perhaps music evolved as a direct auditory pathway toward social and emotional reward centers in the brain.

With regard to the shared evolutionary basis of music with language, it is worth noting that in contrast to music, language does not seem to achieve the same set of evolutionary functions; that is, although language and music both involve connectivity between auditory, motor, and cognitive systems, language has more direct and specific sound-to-meaning mappings, but music more readily establishes aesthetic or emotional connections such as chills (Silvia and Nusbaum, 2011). Thus, language and music may have shared evolutionary origins as a protolanguage (Mithen, 2007), but their divergence led to different evolutionary functions and outcomes.

Successful musical communication depends on an auditory channel through which reward and emotional areas can be accessed. This is consistent with views of music as mixed origins, which posits that music evolved from evolutionarily ancient chill reactions to affiliative sounds (Altenmüller et al., 2013b) that then transform the mind (Patel, 2008). Evolutionarily, the emotional content of sound might have accessed these auditory–reward pathways, which then predisposed the brain toward developing reward sensitivity and thus the need for successful emotional communication. In that regard, results suggest that other species who have connectivity between auditory and reward systems would also be able to enjoy music given the appropriate exposure.

Previous work on congenital amusia has been discussed in terms of its implications on the evolution of music (Patel, 2008); in particular white matter connectivity in congenital amusia supports the hypothesis for a shared basis of music and language (Loui et al., 2009; Loui, 2015). Similarly, white matter connectivity in musical anhedonia informs the evolutionary basis of music on emotion. While reward pathways and auditory perception–action pathways are conventionally seen as separate and dissociable systems in the brain, the present study suggests that they operate in concert, and that this concert of brain systems may be important for the evolution of music: in fact, they may provide support for the MOM as tools to transform the mind (Kleinman, 2015).

Individual differences in structural connectivity between the auditory and reward networks likely represent normal variation in musical reward sensitivity, with some additional patterns that give rise to extreme cases such as musical anhedonia. While increased connectivity between auditory and reward networks is indicative of intense emotional responses to music such as frissons (Harrison and Loui, 2014; Sachs et al., 2016), decreased volume coupled with increased myelination or coherence between specific nodes of these networks reflects the striking lack of specific emotional responses as observed in musical anhedonia. By distinguishing between common variations and rare extremes in individual differences in musical reward sensitivity, the present study attempts to extend the MOM theory by identifying distinct neural pathways through which music might operate as an affective signaling system.

## Author Contributions

PL conceptualized the idea, designed the study, and wrote the first draft of the manuscript. SP, MS, and EP acquired and analyzed the data. YL and TZ contributed DTI data analyses. All authors revised the manuscript and approved the submission.

## Conflict of Interest Statement

The authors declare that the research was conducted in the absence of any commercial or financial relationships that could be construed as a potential conflict of interest.
